# Plate Fixation Versus Intramedullary Nailing for Displaced Clavicular Shaft Fractures: An Updated Meta-Analysis of Randomized Controlled Trials

**DOI:** 10.7759/cureus.93135

**Published:** 2025-09-24

**Authors:** Daniel P Oar, Jason S DeFrancisis, Alex Abouafech, Nicholas Lorenz, Dante DiSilvestro, Alexander Macfarlane

**Affiliations:** 1 Orthopaedic Surgery, Lake Erie College of Osteopathic Medicine, Bradenton, USA; 2 General Surgery, Lake Erie College of Osteopathic Medicine, Bradenton, USA; 3 Medicine, Lake Erie College of Osteopathic Medicine, Bradenton, USA; 4 Orthopaedic Surgery, Trinity Orthopaedics of Western New York, Buffalo, USA

**Keywords:** clavicle fractures, intramedullary nail fixation, meta-analysis, open reduction internal fixation, orif, orthopaedic surgery, orthopedic surgery

## Abstract

Clavicle fractures are a common injury encountered in orthopedic practice, particularly among young, active individuals and older adults with multiple comorbidities. Severely displaced fractures are often treated surgically, with plate fixation (PF) and intramedullary nailing (IMN) representing the two most widely accepted approaches. While both techniques are commonly used, it remains relatively unclear which method yields superior post-operative outcomes. This meta-analysis aims to compare PF and IMN in the surgical management of clavicle fractures by investigating functional recovery scores and perioperative outcomes. A systematic review and meta-analysis of randomized controlled trials (RCTs) was conducted. Studies included in this meta-analysis comprised patients with clavicle fractures treated with either PF or IMN who reported at least one of the following outcomes: Constant-Murley score; Disabilities of the Arm, Shoulder, and Hand (DASH) score; union time; incision length; or operative time. Seven RCTs, involving a total of 455 patients, met the inclusion criteria. The National Institute of Health Quality Assessment Tool was used for risk of bias assessment, and random-effects models were used for data analysis. No significant differences were found between PF and IMN in terms of the Constant-Murley score, DASH score, union time, and operative time, with an overall effect size of -2.67, 4.32, 0.66, and 2.80, respectively. However, IMN was associated with a significantly shorter incision length, with an overall effect size of 6.70, highlighting its minimally invasive nature. Heterogeneity was moderate to high for all outcome measures (I² range: 61.0%-96.2%), indicating appreciable variability between studies. While both techniques are proven to be effective, the choice of surgical method should be tailored to individual patient needs, surgeon experience, and specific fracture characteristics. As variability in outcomes and practice patterns persists, additional high-quality RCTs are needed to clarify long-term differences in functional recovery and surgical outcomes in the operative management of clavicle fractures.

## Introduction and background

Clavicle fractures are among the most frequently encountered injuries in orthopedic practice, representing roughly 2.6% of all fractures in adults and 44% of fractures involving the shoulder girdle [1]. Clavicle fractures tend to occur in younger, active individuals, with 68% of all cases occurring in men [2]. The largest epidemiological subgroup consists of males aged 15-24 years old, who represent 21% of all clavicular fractures [2]. Same-level falls, sports injuries, and traffic accidents constitute the most common mechanisms of injury, while displaced midshaft fractures are the most frequent type of clavicular fracture managed with surgical intervention [2]. The incidence of clavicular fractures also demonstrates a bimodal age distribution, with incidence peaks occurring in individuals younger than 40 and those older than 70 [3]. This secondary peak is seen as increased age and comorbidities lead to more fractures overall in elderly populations, with more than 90% of fractures in the elderly following low-energy falls [4]. Given their prevalence across age groups and their potential to significantly impact shoulder mechanics and patient function, clavicle fractures remain a critical area of orthopedic evaluation and management.

Most clavicle fractures occur in the midshaft diaphyseal region, which accounts for approximately 69%-82% of cases [2]. Distal clavicular fractures constitute a less common subset, representing 12%-26% of cases, while proximal fractures are infrequent, occurring in approximately 2%-6% of patients [2]. This is anatomically supported by the fact that the proximal and distal segments of the clavicle are reinforced by robust muscular and ligamentous attachments, in contrast to the relatively vulnerable midshaft region [2]. Although many patients who have clavicle fractures do achieve adequate healing and functional recovery without surgical interventions, good outcomes, especially with displaced fractures, are not universal [3]. Historically, non-operative management involving immobilization with a sling or figure-of-eight brace has been the mainstay of treatment; however, there is no clear evidence regarding the best technique or duration of immobilization [5]. Current evidence advises non-operative treatment for fractures with a displacement and shortening of less than 2 cm [5]. However, in recent years, there has been a paradigm shift toward operative intervention as recent literature suggests that a subset of midclavicular injuries may warrant surgical treatment to minimize the incidence of non-union, symptomatic malunion, and other suboptimal outcomes [3]. Recent prospective studies have demonstrated non-union rates between 13% and 20% in non-operatively managed cases, with higher risk correlated to factors such as significant displacement, comminuted fracture patterns, and smoking [6]. To reduce the rates of suboptimal outcomes following non-union, operative management may be preferred in some patients [5].

Among the various surgical options for midshaft clavicle fractures, plate fixation (PF) and intramedullary nailing (IMN) remain the two most commonly employed and widely accepted methods [3]. PF facilitates stable compression across the primary fracture line by implementing interfragmentary compression screws to stabilize the plate and bone [3]. Compared to IMN, PF has superior biomechanical strength that offers excellent rotational and length control, allowing early weight bearing of the limb [3]. The main disadvantages of PF are the long skin incision and tissue dissection around the fracture; hardware prominence, which may require plate removal; and possible refracture [3]. Alternatively, IMN has the advantage of preserving more soft tissue, periosteum, and vascular structures around the fracture site, along with possibly lowering infection rates and enhancing fracture callus formation [7]. However, IMN comes with its drawbacks, as some studies suggest it may not offer the same level of rotational stability as PF, particularly in more complex fracture patterns [8]. The question of which technique leads to better functional and operative outcomes remains a subject of ongoing debate. Some surgeons prefer the reliability of plates while others are drawn to the minimally invasive nature of IMN. Functional outcomes are typically assessed using tools like the Constant-Murley (Constant) score and the Disabilities of the Arm, Shoulder, and Hand (DASH) score, both of which help quantify pain, range of motion, strength, and overall upper limb function [9,10]. As well as these functional metrics, additional outcome measures such as fracture union time, incision length, and operative time also factor into treatment decisions and overall patient satisfaction.

Although previous meta-analyses have addressed this research question, their findings were often limited by the relatively small, and often outdated, number of available randomized controlled trials (RCTs) at the time [11]. In recent years, however, a growing number of new RCTs have emerged that have yet to be included in any updated meta-analyses. An updated meta-analysis that includes a comprehensive evaluation of new literature and treatment outcomes is, therefore, both warranted and highly clinically relevant. Despite the influx of new data, a definitive consensus has yet to be established, and clinical practice continues to vary considerably across institutions and providers.

The goal of this meta-analysis is to compare the outcomes of PF versus IMN in the surgical management of clavicular shaft fractures. Functional results using the Constant score and DASH score will be evaluated as primary outcomes while union time, incision length, and operative time will also be evaluated as secondary measures.

## Review

Materials and methods

Reporting

The Preferred Reporting Items for Systematic Reviews and Meta-Analyses (PRISMA) criteria were followed for this review [12].

Research Question

Are there significant differences in Constant score, DASH score, union time, incision length, or operative time between PF and IMN in the surgical management of displaced midshaft clavicle fractures?

Inclusion Criteria

The study population comprised male and female patients of all ages diagnosed with clavicle shaft fractures. Eligible studies included only RCTs. The intervention under investigation was surgical fixation of clavicular shaft fractures using either PF or IMN. Primary outcomes investigated in this study included the Constant score and DASH score, while secondary outcomes included union time, incision length, and operative time. To be included, studies needed to assess at least one of these outcomes at specified follow-up periods and include a standard deviation of group means for adequate data analysis. Additionally, only studies published in English were considered.

Exclusion Criteria

Excluded study designs included systematic reviews, qualitative studies, cross-sectional studies, retrospective studies, case series, case reports, and non-RCTs. Studies published in a language other than English were omitted. Additionally, studies published prior to 2017 were excluded. Studies failing to report at least one outcome of interest and studies with inadequate follow-up were excluded. Non-English studies were excluded due to resource limitations preventing accurate translation and data extraction.

Search Strategy

A comprehensive search of publications through August 2025 was conducted in the Google Scholar and PubMed databases. Search terms included “IMN”, AND “PF”, AND “clavicle fractures”, AND “Constant score”, OR “DASH score”, OR “operative time”, OR “union time”, OR “incision length”. This search resulted in 3,950 results, which were subsequently screened in a sequential manner.

Data Sources

Manual searches of relevant systematic reviews and meta-analyses were conducted to identify other studies not listed under our original database search in May 2025.

Study Selection

A total of 3,950 records were screened for eligibility based on predefined inclusion and exclusion criteria as outlined in previous sections. Three thousand nine hundred thirty-eight results were screened out for being published before 2017, being unrelated to PF or IMN of clavicle fractures, or not being a RCT. Search results were additionally screened for duplicates. This resulted in 12 full-text articles available for further assessment. Five of these full-text articles were additionally excluded for either inadequate follow-up (n = 1) or inadequate data reporting (n = 4). Seven full-text articles were included in our final data analysis (n = 7). No disagreements were found between reviewers (D.O. and J.D.).

Data Extraction

Data related to sample size, study design, confidence intervals, standard deviation, Constant scores, DASH scores, union time, incision length, operative time, and follow-up period were extracted from each study. Union times reported in months were converted to weeks, and incision lengths reported in millimeters were converted to centimeters, where appropriate, for consistent data analysis.

Quality Assessment

In order to reduce potential bias, inclusion was limited to RCTs. Bias assessment was performed using the NIH Study Quality Assessment Tool and funnel plots [13].

Statistical Analysis

Data analysis and graph production were performed using RStudio (v4.4.1, R Core Team, 2024) and the meta package version 7.0-0. Forest plots were generated for each outcome variable measured to present each study's mean value and associated confidence interval. A random-effects model with 95% confidence intervals was employed to evaluate heterogeneity. Heterogeneity was assessed using the I² and Tau² statistics to evaluate variation across studies.

Assessment of Results

This meta-analysis includes calculations using both the random-effects and common-effects models. Subsequent sections of this manuscript focus exclusively on the random-effects model, as it represents the most appropriate framework for analyzing the study results [14,15].

Results

Literature Search

**Figure 1:**
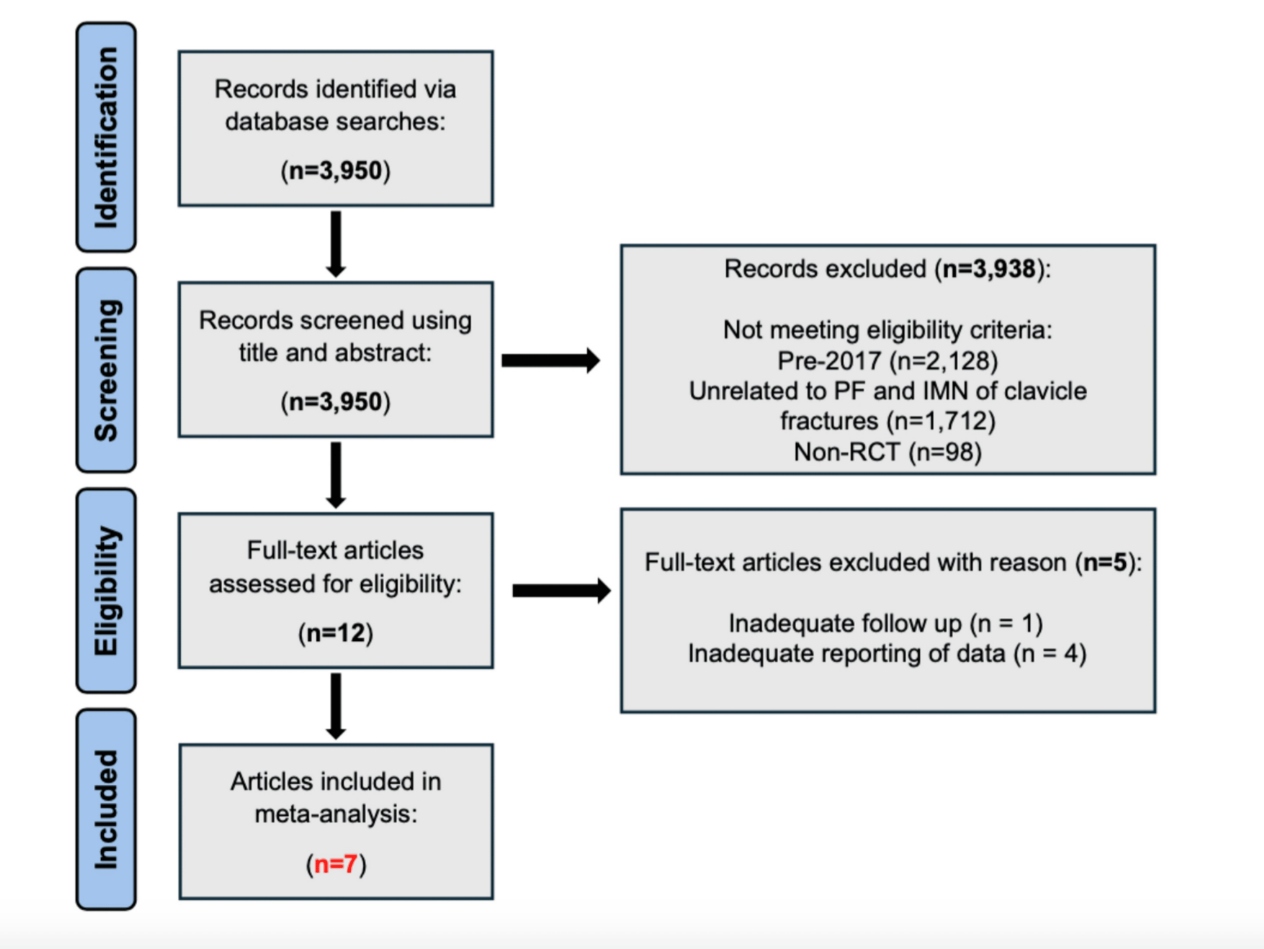
A PRISMA flow chart displaying the literature search and selection process n: number; PF: plate fixation; IMN: intramedullary nailing; non-RCT: non-randomized controlled trial; PRISMA: Preferred Reporting Items for Systematic Reviews and Meta-Analyses

The total number of studies found in our initial literature search was 3,950. After screening out any studies that did not meet all of our eligibility criteria, seven studies were identified and included in our data analysis (Figure 1) [16-22].

Characteristics of Studies Included

Seven studies were included in this meta-analysis [16-22]. Study characteristics are summarized in Table 1.

**Table 1:** Summary of literature search and study characteristics included in this statistical analysis RCT: randomized controlled trial; PF: plate fixation; IMN: intramedullary nailing; DASH: Disabilities of the Arm, Shoulder, and Hand; Constant: Constant-Murley

Study	Total sample size (n)	PF group size (n)	IMN group size (n)	Study design	Treatment	Outcomes measured
Calbiyik et al., 2017 [16]	75	40	35	RCT	PF and IMN of clavicle fractures	Constant score, DASH score, union time, incision length, operative time
Pal et al., 2018 [17]	66	33	33	RCT	PF and IMN of clavicle fractures	Union time, incision length
Sharma et al., 2019 [18]	50	25	25	RCT	PF and IMN of clavicle fractures	Union time, incision length, operative time
King et al., 2019 [19]	72	37	35	RCT	PF and IMN of clavicle fractures	Constant score, DASH score, incision length, operative time
Yadav et al., 2022 [20]	62	31	31	RCT	PF and IMN of clavicle fractures	Constant score
Nama et al., 2024 [21]	100	50	50	RCT	PF and IMN of clavicle fractures	Union time, incision length, operative time
Saha et al., 2025 [22]	30	15	15	RCT	PF and IMN of clavicle fractures	DASH score, union time

Risk-of-Bias Assessment

The NIH Study Quality Assessment Tool was utilized for risk of bias assessment [13]. Two reviewers (D.O. and J.D.) independently conducted the quality assessment. If a disagreement arose between reviewers on any of the provided questions, a third reviewer (A.A.) determined a tie-breaking judgment. Scoring was conducted by awarding one point for “yes” responses and zero points for “no” responses to the NIH Study Quality Assessment Tool questions. An article was deemed “poor” if it was awarded 0-4 points total, “fair” if it was awarded 5-9 points total, and “good” if it was awarded 10-14 points total. Results from this risk-of-bias assessment can be found in Table 2. The risk of bias in Calbiyik et al. was “good”; in Pal et al., it was “fair”; in Sharma et al., it was “fair”; in King et al., it was “fair”; in Yadav et al., it was “fair”; in Nama et al., it was “fair”; and in Saha et al., it was “fair” (Table 2) [16-22]. Funnel plots were also used to highlight the risk of publication bias for each outcome measure.

**Table 2:** Risk-of-bias quality assessment using NIH criteria questions Items 1-14 represent the 14 criteria questions used in the NIH Quality Assessment of Controlled Intervention Studies. A "yes" or "no" answer is provided for each question [13]. Bias assessment was conducted using the NIH Quality Assessment of Controlled Intervention Studies, which measures the methodological quality of each study [13]. F: fair; G: good; N: no; Y: yes

Study	Bias ruling	Total score (out of 14)	1	2	3	4	5	6	7	8	9	10	11	12	13	14
Calbiyik et al., 2017 [16]	G	10	Y	Y	N	N	N	Y	Y	Y	Y	Y	Y	Y	N	Y
Pal et al., 2018 [17]	F	8	Y	N	N	N	N	Y	Y	Y	Y	Y	Y	N	N	Y
Sharma et al., 2019 [18]	F	8	Y	N	N	N	N	Y	Y	Y	Y	Y	Y	N	N	Y
King et al., 2019 [19]	F	9	Y	Y	N	N	N	Y	Y	Y	Y	Y	Y	Y	N	N
Yadav et al., 2022 [20]	F	9	Y	N	N	N	N	Y	Y	Y	Y	Y	Y	N	Y	Y
Nama et al., 2024 [21]	F	8	Y	N	N	N	N	Y	Y	Y	Y	Y	Y	N	N	Y
Saha et al., 2025 [22]	F	7	Y	N	N	N	N	N	Y	Y	Y	Y	Y	N	N	Y

Findings

This meta-analysis included seven RCTs (Figure 1). Across all seven trials, a total of 455 patients with clavicle fractures were considered. Of these, 231 participants were treated with PF and 224 with IMN. Shared outcomes across the seven RCTs were extracted and categorized as primary or secondary outcomes. The primary outcomes of this meta-analysis consisted of the Constant score and DASH score, while secondary outcomes consisted of union time, incision length, and operative time. All outcomes were assessed and evaluated with a random-effects model.

**Figure 2:**
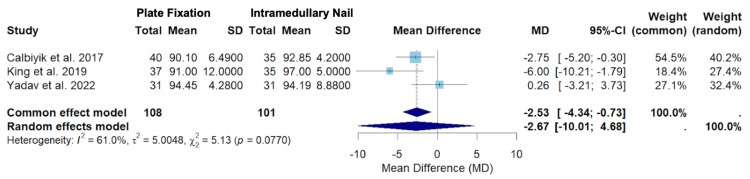
A forest plot of the mean difference in Constant score between PF and IMN SD: standard deviation; CI: confidence interval; PF: plate fixation; IMN: intramedullary nailing Studies included: Calbiyik et al., King et al., and Yadav et al. [16,19,20]

**Figure 3:**
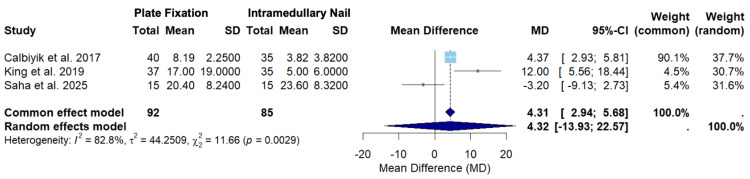
A forest plot of the mean difference in DASH scores between PF and IMN SD: standard deviation; CI: confidence interval; DASH: Disabilities of the Arm, Shoulder, and Hand; PF: plate fixation; IMN: intramedullary nailing Studies included: Calbiyik et al., King et al., and Saha et al. [16,19,22]

The results for the random-effects models for the primary outcomes of Constant score and DASH score can be found in Figures 2, 3.

Figure 2 displays the random-effects model for Constant scores at the 12-month follow-up among Calbiyik et al., King et al., and Yadav et al. [16,19,20]. The overall mean difference using the random-effects model was -2.67 (-10.01; 4.68) (Figure 2). As the confidence interval includes zero, the random-effects model suggests that there was no statistically significant difference in Constant scores at the 12-month follow-up between clavicle fracture patients treated with PF versus IMN (Figure 2). This effect leans toward a higher Constant score in the IMN group, but this difference fails to reach statistical significance. The effect sizes varied appreciably between the studies. Calbiyik et al. contributed the highest weight in the random-effects model (40.2%) (Figure 2). Calbiyik et al. also had the highest precision, seen by the narrowest confidence interval range (-5.20; -0.30) (Figure 2). The overall heterogeneity (I2 = 61.0%) suggests high heterogeneity, although the test for heterogeneity was not statistically significant (p = 0.0770) (Figure 2) [23,24]. This indicates that the hypothesis of no heterogeneity may not be rejected (Figure 2).

Figure 3 displays the random-effects model for DASH scores among Calbiyik et al., King et al., and Saha et al. [16,19,22]. The overall mean difference using the random-effects model was 4.32 (-13.93; 22.57) (Figure 3). Since the confidence interval crosses zero, the random-effects model suggests that there was no statistically significant difference in DASH scores between clavicle fracture patients treated with PF versus IMN (Figure 3). This effect leans toward a higher DASH score in the PF group, but this difference fails to reach statistical significance. The effect sizes varied between the studies. Calbiyik et al. contributed the highest weight in the random-effects model (37.7%) (Figure 3). Calbiyik et al. also had the highest precision, seen by the narrowest confidence interval range (2.93; 5.81) (Figure 3). The overall heterogeneity (I2 = 82.2%) suggests substantial heterogeneity, and the test for heterogeneity was statistically significant (p = 0.0029), suggesting rejection of the null hypothesis of homogeneity (Figure 3).

**Figure 4:**
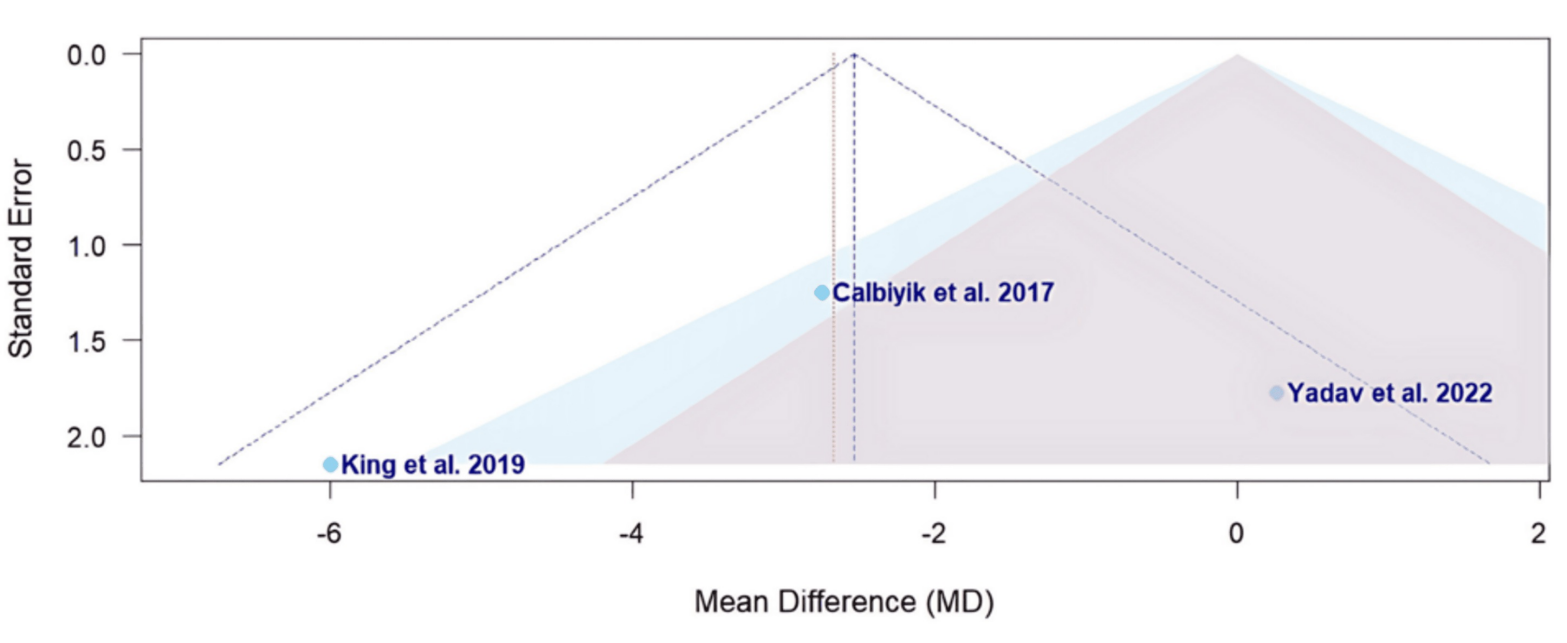
A funnel plot of publication bias for Calbiyik et al., King et al., and Yadav et al. in regard to the Constant score Studies included: Calbiyik et al., King et al., and Yadav et al. [16,19,20]

**Figure 5:**
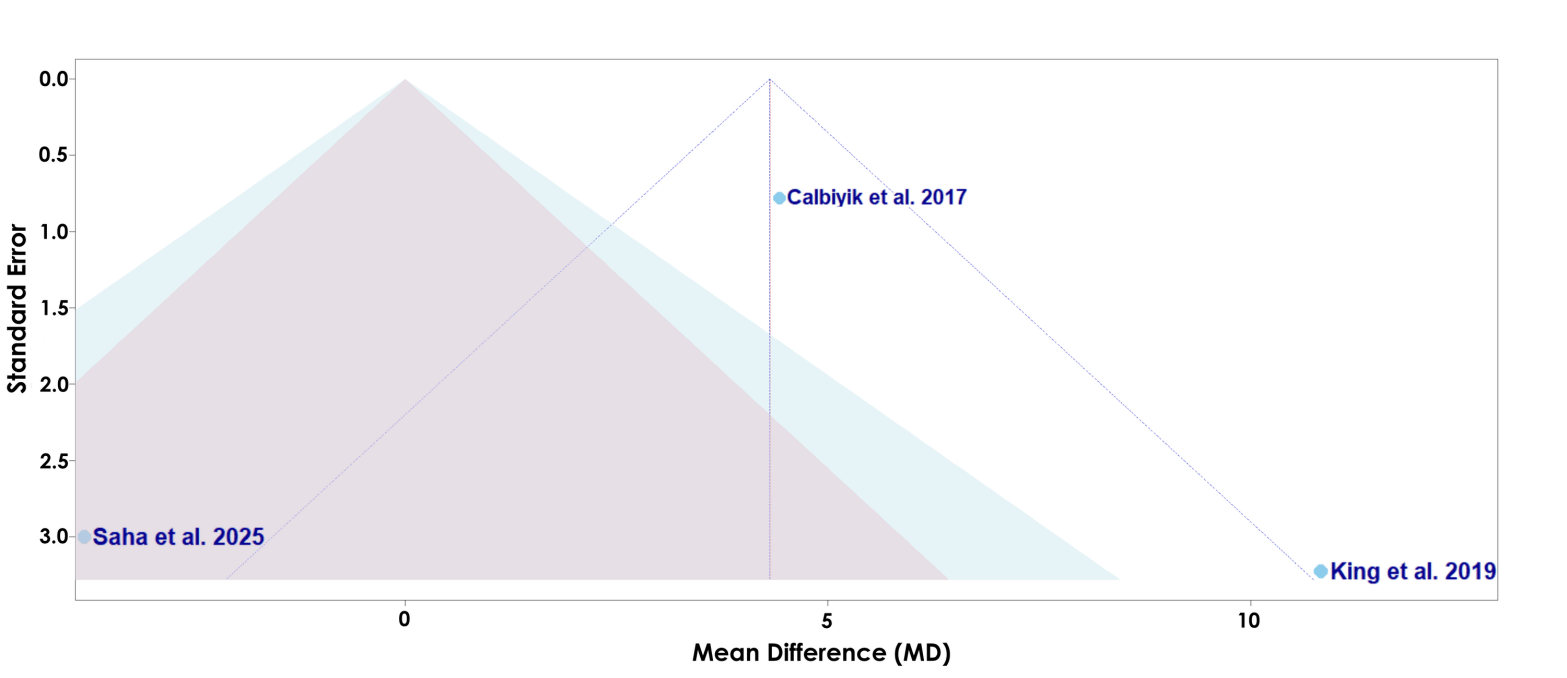
A funnel plot of publication bias for Calbiyik et al., King et al., and Saha et al. in regard to the DASH score Studies included: Calbiyik et al., King et al., and Saha et al. [16,19,22]

The funnel plots visualized in Figures 4, 5 assess the risk of bias of each study for the primary outcomes of Constant score and DASH score, respectively.

The funnel plot visualized in Figure 4 assesses the potential risk of bias among the studies evaluating the Constant scores [16,19,20]. Calbiyik et al. is located near the vertical midline with a lower standard error, suggesting a lower likelihood of bias (Figure 4) [16]. Conversely, King et al. appears as the lowest point with a higher standard error, suggesting a higher likelihood of bias (Figure 4) [19].

The funnel plot presented in Figure 5 assesses the potential risk of bias across the studies assessing DASH scores [16,19,22]. Calbiyik et al. is positioned closest to the midline with a lower standard error, representing the lowest risk of bias or variability (Figure 5) [16]. In contrast, King et al. and Saha et al. both deviate substantially from the midline and appear lower on the plot with high standard errors, representing a higher risk of bias or variability (Figure 5) [19,22].

**Figure 6:**
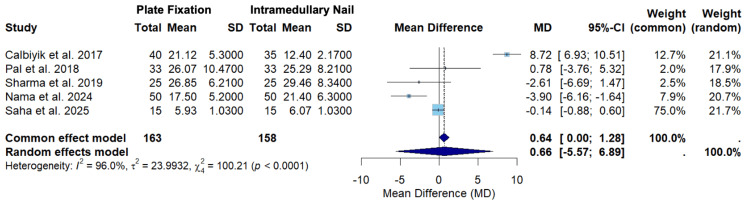
A forest plot of the mean difference in union time between PF and IMN SD: standard deviation; CI: confidence interval; PF: plate fixation; IMN: intramedullary nailing Studies included: Calbiyik et al., Pal et al., Sharma et al., Nama et al., and Saha et al. [16-18,21,22]

Figure 6 displays the random-effects model for a secondary outcome of union time among Calbiyik et al., Pal et al., Sharma et al., Nama et al., and Saha et al. [16-18,21,22].

Figure 6 displays the random-effects model for union time among Calbiyik et al., Pal et al., Sharma et al., Nama et al., and Saha et al. [16-18,21,22]. The overall mean difference using the random-effects model was 0.66 (-5.57; 6.89) (Figure 6). Since the confidence interval crosses zero, the random-effects model suggests that there was no statistically significant difference in union time between clavicle fracture patients treated with PF versus IMN (Figure 6). This effect leans toward a greater union time in the PF group, but this difference fails to reach statistical significance. The effect sizes varied between the studies. Saha et al. contributed the highest weight in the random-effects model (21.7%) (Figure 6). Saha et al. also had the highest precision, seen by the narrowest confidence interval range (-0.88; 0.60) (Figure 6). The overall heterogeneity (I2 = 96.0%) suggests substantial heterogeneity, and the test for heterogeneity was statistically significant (p < 0.0001), suggesting rejection of the null hypothesis of homogeneity (Figure 6).

**Figure 7:**
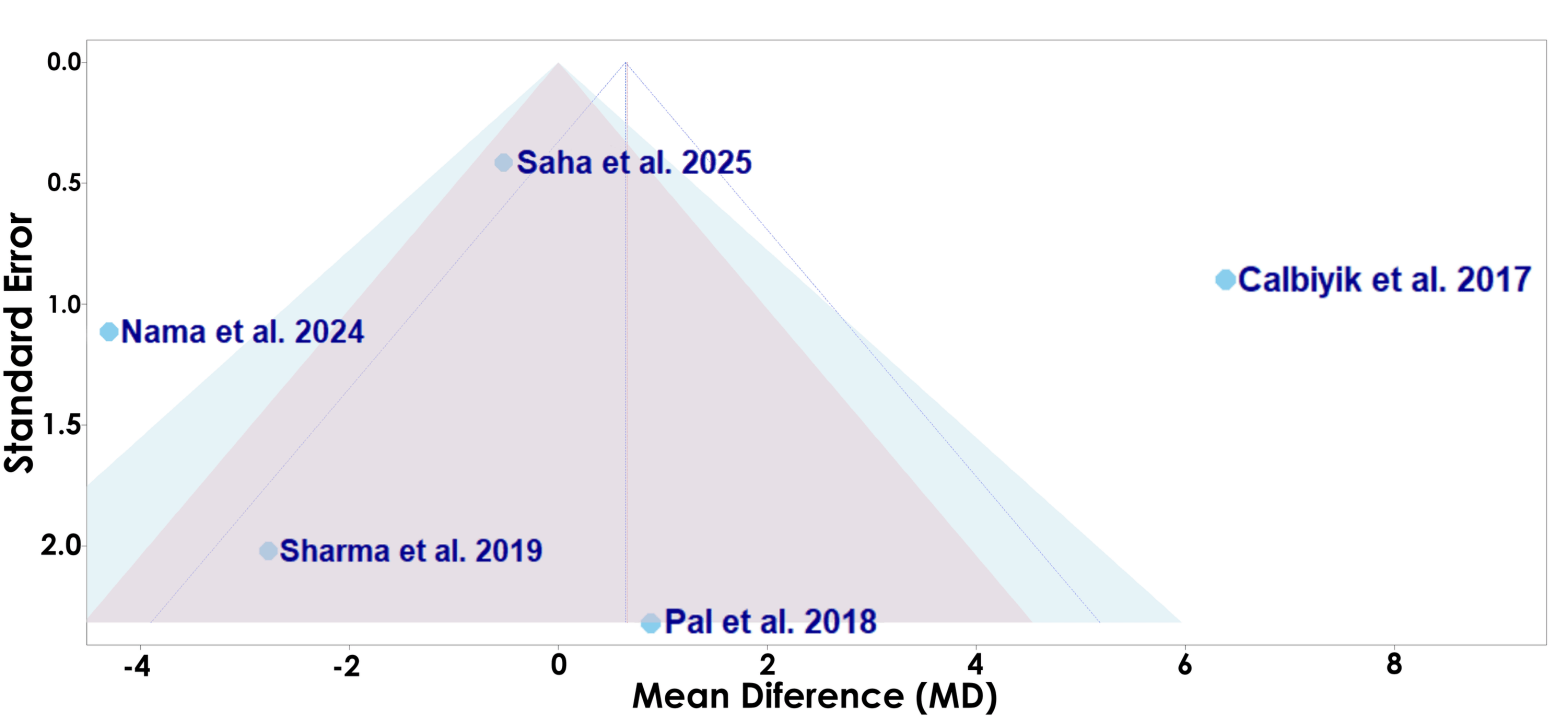
A funnel plot of publication bias for Calbiyik et al., Pal et al., Sharma et al., Nama et al., and Saha et al. in regard to union time Studies included: Calbiyik et al., Pal et al., Sharma et al., Nama et al., and Saha et al. [16-18,21,22]

The funnel plot seen in Figure 7 assesses the risk of bias of each study for the secondary outcome of union time.

The funnel plot displayed in Figure 7 assesses the potential risk of bias among the studies evaluating the union time [16-18,21,22]. Saha et al. is located near the vertical midline with a lower standard error, suggesting a lower likelihood of bias (Figure 7) [22]. Conversely, Pal et al. appears as the lowest point with a higher standard error, suggesting a higher likelihood of bias, yet falls near the vertical midline, indicating the effect estimate should be interpreted with caution (Figure 7) [17].

**Figure 8:**
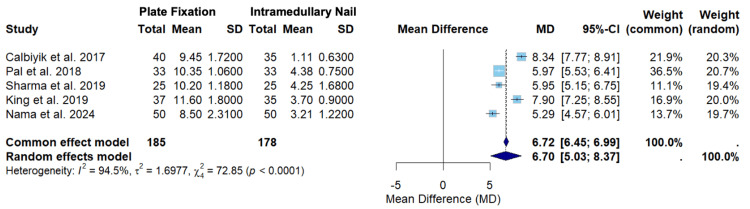
A forest plot of the mean difference in incision length between PF and IMN SD: standard deviation; CI: confidence interval; IMN: intramedullary nailing; PF: plate fixation Studies included: Calbiyik et al., Pal et al., Sharma et al., King et al., and Nama et al. [16-19,21]

Figure 8 displays the random-effects model for a secondary outcome of incision length among Calbiyik et al., Pal et al., Sharma et al., King et al., and Nama et al. [16-19,21].

Figure 8 displays the random-effects model for incision length among Calbiyik et al., Pal et al., Sharma et al., King et al., and Nama et al. [16-19,21]. The overall mean difference using the random-effects model was 6.70 (5.03; 8.37) (Figure 8). Since the confidence interval does not cross zero, the random-effects model suggests that there was a statistically significant difference in incision length between clavicle fracture patients treated with PF versus IMN, with IMN showing a significantly shorter length of incision (Figure 8). The effect sizes varied between the studies. Pal et al. contributed the highest weight in the random-effects model (20.7%) (Figure 8). Pal et al. also had the highest precision, seen by the narrowest confidence interval range (5.53; 6.41) (Figure 8). The overall heterogeneity (I2 = 94.5%) suggests substantial heterogeneity, and the test for heterogeneity was statistically significant (p < 0.0001), suggesting rejection of the null hypothesis of homogeneity (Figure 8).

**Figure 9:**
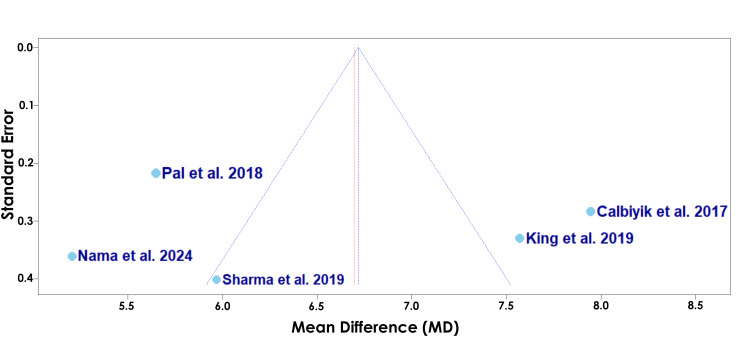
A funnel plot of publication bias for Calbiyik et al., Pal et al., Sharma et al., King et al., and Nama et al. in regard to incision length Studies included: Calbiyik et al., Pal et al., Sharma et al., King et al., and Nama et al. [16-19,21]

The funnel plot displayed in Figure 9 assesses the risk of bias of each study for the secondary outcome of incision length.

The funnel plot displayed in Figure 9 assesses the potential risk of bias among the studies evaluating incision length [16-19,21]. Pal et al. is located relatively close to the vertical midline with a lower standard error, suggesting a lower likelihood of bias (Figure 9) [17]. Conversely, Sharma et al. appears as the lowest point with a higher standard error, suggesting a higher likelihood of bias, yet falls relatively close to the vertical midline, indicating the effect estimate should be interpreted with caution (Figure 9) [18]. Nama et al. appears as another relatively low point with a high standard error and also strays far from the vertical midline, suggesting a higher likelihood of bias and variability [21].

**Figure 10:**
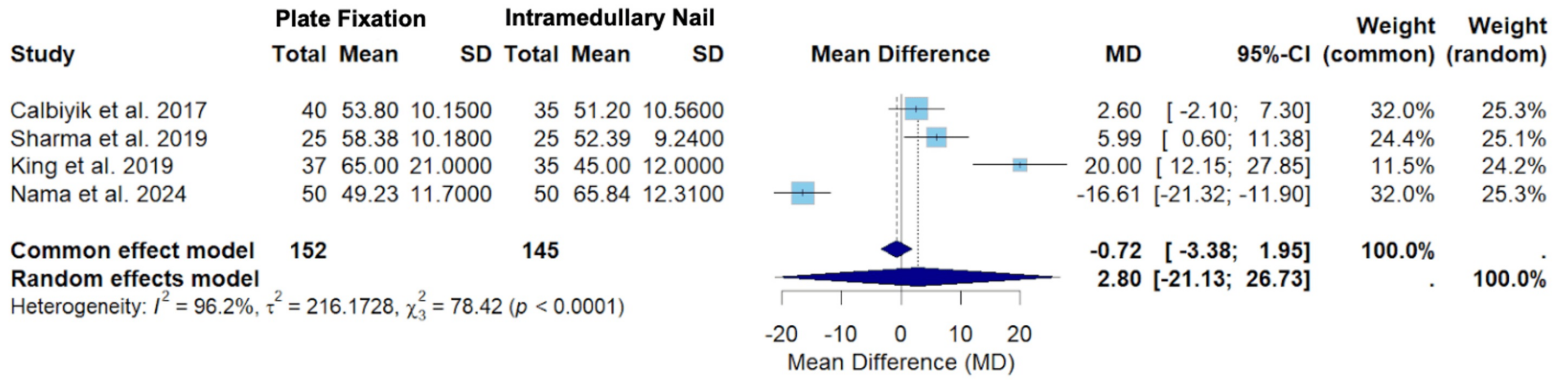
A forest plot of the mean difference in operative time between PF and IMN SD: standard deviation; CI: confidence interval; PF: plate fixation; IMN: intramedullary nailing Studies included: Calbiyik et al., Sharma et al., King et al., and Nama et al. [16,18,19,21]

Figure 10 displays the random-effects model for a secondary outcome of operative time among Calbiyik et al., Sharma et al., King et al., and Nama et al. [16,18,19,21].

Figure 10 displays the random-effects model for operative time among Calbiyik et al., Sharma et al., King et al., and Nama et al. [16,18,19,21]. The overall mean difference using the random-effects model was 2.80 (-21.13; 26.73) (Figure 10). Since the confidence interval crosses zero, the random-effects model suggests that there was no statistically significant difference in operative time between clavicle fracture patients treated with PF versus IMN (Figure 10). This effect leans toward a greater operative time in the PF group, but this difference fails to reach statistical significance. The effect sizes varied only slightly between the studies. Calbiyik et al. and Nama et al. equally contributed the highest weight in the random-effects model (25.3%) (Figure 10). Calbiyik et al. had the highest precision, seen by the narrowest confidence interval range (-2.10; 7.30) (Figure 10). The overall heterogeneity (I2 = 96.2%) suggests substantial heterogeneity, and the test for heterogeneity was statistically significant (p < 0.0001), suggesting rejection of the null hypothesis of homogeneity (Figure 10).

**Figure 11:**
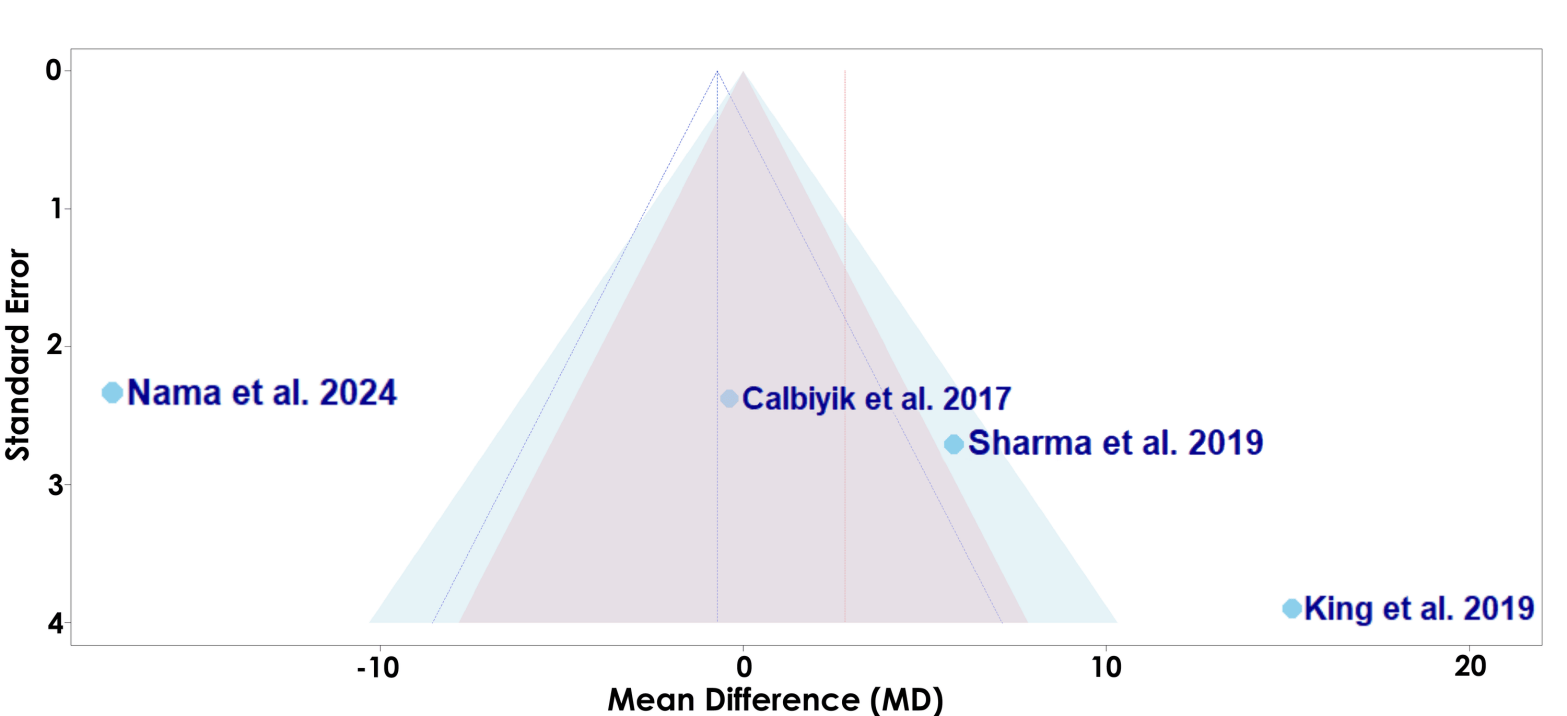
A funnel plot of publication bias for Calbiyik et al., Sharma et al., King et al., and Nama et al. in regard to operative time Studies included: Calbiyik et al., Sharma et al., King et al., and Nama et al. [16,18,19,21]

The funnel plot shown in Figure 11 assesses the risk of bias of each study for the secondary outcome of operative time.

The funnel plot displayed in Figure 11 demonstrates the potential risk of bias among studies evaluating the operative time [16,18,19,21]. Calbiyik et al. is located closest to the vertical midline with a relatively low standard error, suggesting a lower likelihood of bias (Figure 11) [16]. Conversely, King et al. appears as the lowest point with a higher standard error, suggesting a higher likelihood of bias (Figure 11) [19].

Discussion

This meta-analysis analyzed data from seven RCTs to compare the effectiveness of PF versus IMN in the management of displaced midshaft clavicle fractures. Our results demonstrated no statistically significant difference in functional outcomes between the two fixation techniques. Both Constant score and DASH score were similar at follow-up, with no statistically significant difference, suggesting that either approach provides relatively equivalent long-term recovery in terms of shoulder function and disability (Figures 2, 3). Secondary outcomes of union time and operative time also showed no statistically significant differences between techniques (Figures 6, 10). However, incision length was significantly shorter in the IMN group, supporting its advantage as a less invasive technique (Figure 8).

These findings are consistent with trends reported in previous meta-analyses, which have similarly concluded that PF and IMN result in comparable functional outcomes [11,25]. Some of these studies have noted additional potential advantages of IMN, such as reduced operative time, faster union time, fewer infections, and shorter hospital stays [25,26]. Our meta-analysis did not replicate all of these findings, which may reflect differences in study selection criteria, outcome definitions, follow-up intervals, or variability in surgical techniques across included trials. Some prior meta-analyses incorporated a larger quantity of older trials with a broader range of outcome measures, while others included studies with different definitions of union or timing of postoperative assessments [11,25,26]. Additionally, surgeon experience with either technique may contribute to variations in operative duration or complication rates. Despite these differences, the overall evidence continues to support the functional equivalence of the two techniques, with a possible cosmetic and procedural benefit favoring IMN.

From a clinical standpoint, these results suggest that both PF and IMN are valid surgical options for appropriately selected patients with midshaft clavicle fractures. Given the similar functional outcomes, surgical choice may depend more on fracture pattern, surgeon familiarity, and patient preferences. IMN offers the benefit of smaller incisions and potentially reduced soft tissue disruption, which may be particularly appealing in younger or more active patients who value cosmetic appearance [3,7]. PF, on the other hand, may offer greater versatility and stability in more complex fracture configurations [27].

This study possesses several methodological strengths. Restricting inclusion criteria to RCTs published in 2017 or later ensured the incorporation of data representative of modern surgical techniques and practice standards. The use of multiple clinically meaningful outcomes, both functional and procedural, allowed for a comprehensive comparison between these two surgical interventions. Furthermore, the analysis adhered to PRISMA reporting guidelines and employed rigorous statistical methodology, including random-effects modeling and an assessment of publication bias [12].

Nevertheless, several limitations should be acknowledged. High heterogeneity was present in many of the secondary outcomes, including union time, incision length, and operative time. This likely reflects underlying variability in patient populations, fracture complexity, implant types, and perioperative protocols across trials (Figures 6, 8, 10). Additionally, although all included studies were RCTs, most were rated as “fair” quality, and few provided sufficient detail on their randomization methods, allocation concealment, or blinding (Table 2). Consequently, all studies analyzed cannot be confidently categorized as high-quality RCTs. Follow-up durations for the DASH score were also not consistent across studies, ranging from 3 to 12 months, limiting direct comparability of this functional outcome measure (Figure 3). Publication bias may also be present, as suggested by asymmetries in funnel plots (Figures 4, 5, 7, 9, 11). However, results should be interpreted with caution, given the relatively small number of included studies that may limit statistical power.

Future studies should prioritize enhancing methodological rigor by ensuring comprehensive disclosure of randomization and blinding protocols, standardizing follow-up intervals, and employing consistent, validated outcome measures. Larger, multicenter trials would improve statistical power and generalizability. Studies that incorporate patient-reported outcomes such as satisfaction, cosmetic perception, and return to activity, as well as cost-effectiveness analyses, would offer a more complete understanding of the relative merits of each approach. Additionally, stratifying patients based on fracture morphology and baseline characteristics may help refine surgical decision-making for specific patient populations.

Both PF and IMN appear to provide comparable functional outcomes in the treatment of midshaft clavicle fractures. While differences in union time and operative time were not statistically significant, IMN demonstrated a meaningful reduction in incision length, highlighting its minimally invasive advantage. These findings support the continued use of both techniques, with patient and surgeon-specific factors guiding the optimal choice of fixation for each individual patient.

## Conclusions

This meta-analysis found no statistically significant differences between PF and IMN in terms of functional recovery, union time, or operative time for displaced midshaft clavicle fractures. Both techniques provided comparable outcomes for the Constant score and DASH score, suggesting equivalent effectiveness in restoring shoulder function. The only significant difference observed was a shorter incision length with IMN, reflecting its minimally invasive nature. Given the overall similarity in results, both surgical options remain valid approaches for appropriately selected patients. The choice of fixation method should be guided by individual patient characteristics, fracture configuration, and surgeon expertise. Further high-quality RCTs should ensure rigorous methodology with clear randomization and blinding, standardized follow-up, multicenter design for increased generalizability, and stratification by fracture characteristics to better guide surgical decision-making for the operative management of clavicle fractures.
